# An Evaluation of Sex-Specific Pharmacokinetics and Bioavailability of Kokusaginine: An In Vitro and In Vivo Investigation

**DOI:** 10.3390/ph17081053

**Published:** 2024-08-09

**Authors:** Kaiqi Shang, Chengyu Ge, Yindi Zhang, Jing Xiao, Shao Liu, Yueping Jiang

**Affiliations:** 1 Department of Pharmacy, Xiangya Hospital, Central South University, Changsha 410008, China; 15186814762@163.com (K.S.); gechengyu0408@163.com (C.G.); 228112404@csu.edu.cn (Y.Z.); 2 National Clinical Research Center for Geriatric Disorders, Xiangya Hospital, Central South University, Changsha 410008, China; 3 The Hunan Institute of Pharmacy Practice and Clinical Research, Changsha 410008, China; 4 Hunan Institute for Drug Control, Changsha 410001, China; 15367917800@189.cn; 5 College of Pharmacy, Changsha Medical University, Changsha 410219, China

**Keywords:** kokusaginine, pharmacokinetic, bioavailability, sex difference, rat liver microsomes

## Abstract

Kokusaginine is a bioactive ingredient extracted from *Ruta graveolens* L., which has a range of biological activities. Its pharmacokinetic (PK) properties are particularly important for clinical applications; however, they have not been fully elucidated. In addition, the effect of sex differences on drug metabolism is increasingly being recognized, but most studies have ignored this important factor. This study aims to fill this knowledge gap by taking an in-depth look at the PK properties of kokusaginine and how gender affects its metabolism and distribution in the body. It also lays the foundation for clinical drug development. In this study, a sensitive ultra-high-performance liquid chromatography (UPLC) method was developed and validated for quantifying kokusaginine in Sprague Dawley (SD) rat plasma and tissue homogenates. Metabolic stability was evaluated in vitro using gender-specific liver microsomes. Innovatively, we incorporated sex as a variable into both in vitro and in vivo PK studies in SD rats, analyzing key parameters with Phoenix 8.3.5 software. The developed UPLC method demonstrated high sensitivity and precision, essential for PK analysis. Notably, in vitro studies revealed a pronounced sex-dependent metabolic variability (*p* < 0.05). In vivo, gender significantly affected the Area Under the Moment Curve (AUMC)_(0-∞)_ of the plasma PK parameter (*p* < 0.05) and the AUMC_(0-t)_ of brain tissue (*p* < 0.0001), underscoring the necessity of sex-specific PK assessments. The calculated absolute bioavailability of 71.13 ± 12.75% confirmed the favorable oral absorption of kokusaginine. Additionally, our innovative tissue-plasma partition coefficient (K_p_) analysis highlighted a rapid and uniform tissue distribution pattern. This study presents a sex-inclusive PK evaluation of kokusaginine, offering novel insights into its metabolic profile and distribution. These findings are instrumental for informing clinical medication practices, dosage optimization, and a nuanced understanding of drug efficacy and safety in a sex-specific context.

## 1. Introduction

Traditional Chinese medicine has been an important resource for drug discovery, highlighting numerous potentially active lead compounds that often exhibit a variety of important biological activities [1,2,3]. *Ruta graveolens* L. has a place in traditional Chinese medicine for its unique medicinal properties; it contains a variety of bioactive ingredients and is widely used to treat inflammation, pain, and certain skin diseases [4,5,6,7]. Kokusaginine is a furanoquinoline alkaloid isolated from *Ruta graveolens* L. Recent studies have shown that it has significant anti-inflammatory, antibacterial, and anti-insect activities [8,9]. Our previous studies further explored the potential role of kokusaginine in the clinical treatment of renal fibrosis, showing that it can effectively inhibit the activation of the PI3K/AKT signaling pathway, thereby preventing the formation of renal fibrosis in vivo and in vitro [10]. In addition, a study by Deniz et al. reported the inhibitory effect of kokusaginine on acetylcholinesterase (AChE) and butylcholinesterase (BChE) [11], which highlights the possibility of its potential as a novel anti-Alzheimer’s disease drug. Other studies have also shown that kokusaginine can not only inhibit the proliferation of cancer cells [12] but also show an effective inhibitory effect on MCF-7/ADR of multi-drug-resistant breast cancer cells [13] and inhibit the function of P-glycoprotein (P-gp). The antiplasmodium activity of kokusaginine has also been reported [14]. Later, Valdez et al. confirmed its anti-parasitic effects [8]. These findings suggest that kokusaginine has a wide range of pharmacological activities and may be a promising candidate for the treatment of diseases as diverse as renal fibrosis, neurodegenerative diseases, cancer, malaria, and parasitic diseases.

In view of the significant pharmacological potential of kokusaginine, which has been confirmed by a number of studies [15,16], an in-depth PK study of kokusaginine is particularly critical as it is an indispensable part of drug development and clinical application. PK studies are not only able to reveal the level of absorption and tissue distribution of kokusaginine in vivo but are also critical for evaluating its potential as a drug candidate. However, the in vivo PKs, tissue distribution, and bioavailability of kokusaginine have not yet been reported in the literature, and this information is essential to fully understanding its clinical application prospects. Liver metabolic stability is a key factor affecting the oral bioavailability and plasma concentration of compounds, so their precise measurement in the early stages of drug development is critical. Preliminary metabolic stability assessment in the liver microsomal system provides us with information about the metabolic stability of compounds in vivo, which is important for predicting their PK behavior and preclinical evaluation.

The objective of this study was to conduct a comparative PK analysis of kokusaginine, both in vitro and in vivo, to elucidate the differences and similarities in its metabolic behavior. The internal standard method was selected for quantitative analysis. Because the chemical structure of dictamine is similar to that of kokusaginine and its chromatographic peak position is close to and fully separated from that of kokusaginine, dictamine was selected as the internal standard for analysis. In designing the experiment, we took into account that gender may have a significant impact on the metabolism, distribution, excretion, and effects of the drug. Therefore, we used liver microsomes from rats of different genders in in vitro experiments to assess the effect of sex on the metabolic stability of kokusaginine. This out-of-body assessment provides a basis for understanding the inherent metabolic characteristics of kokusaginine, independent of the physiological variable of sex. Subsequently, we expanded our study to include rats of different genders, and the drug was administered orally and by tail vein injection. The dosage was set based on a previous pharmacological experiment performed in our research group. In a previous experiment [10], kokusaginine (10, 20, and 40 mg/kg) was administered orally to mice in the treatment group, and the equivalent doses for rats were 7, 14, and 28 mg/kg by body surface area. Therefore, doses of 28 mg/kg for oral administration and 7 mg/kg for intravenous administration were selected in this study as they had the best therapeutic effect. This approach allowed us to determine the PKs and tissue distribution of kokusaginine in the complex biological environment of the organism. In the PK evaluation of kokusaginine, certain organs were selected to comprehensively evaluate its clinical potential and safety. The liver and kidneys were included in this study because of their critical metabolic and excretory functions [17,18], while kokusaginine may have specific effects on the kidneys [10]. The brain was selected to assess kokusaginine’s ability to penetrate the blood-brain barrier and provide a basis for clinical studies. The evaluation of the heart and spleen reflects our concern about the safety of the drug, particularly its direct impact on cardiac function and its regulatory effect on the immune system [19]. In addition, given the anti-inflammatory properties of kokusaginine, the examination of the lungs helps in exploring its application in anti-inflammatory therapy [20]. These combined considerations ensure an in-depth understanding of the many facets of kokusaginine. By comparing the in vitro metabolic stability with the in vivo PK parameters, we were able to assess the absolute bioavailability of kokusaginine and its distribution across key tissues such as the heart, liver, spleen, lung, kidney, and brain tissues.

This systematic study of the PK properties of kokusaginine not only bridges the gap between in vitro and in vivo assessments but also offers a valuable reference for further exploration of its clinical potential. The comparative analysis underscores the importance of understanding the behavior of compounds such as kokusaginine in different biological contexts, which is crucial for the advancement of its therapeutic applications.

## 2. Results

### 2.1. Optimization of Sample Preparation

We optimized the sample preparation process. To remove proteins and other endogenous components that may cause interference, the sample was pre-treated by protein precipitation, liquid-liquid extraction, and a combination of the two methods while taking into account the chemical properties of kokusaginine. The results showed that the recovery rate of protein precipitation with acetonitrile and extraction with ethyl acetate was higher, so it was chosen as the pre-treatment method.

### 2.2. Optimization of UPLC

To optimize the detection conditions of kokusaginine, different chromatographic conditions were evaluated. The chromatographic performance of different columns, including the Agilent ZORBAXSB-C18 (4.6 × 250 mm, 5 μm) and HS12S05-2546WT YMC Hydrosphere C18 (4.6 × 250 mm, 5 μm), was evaluated and compared. Kokusaginine showed a longer retention time on the latter, with more baseline noise. Therefore, the Agilent ZORBAXSB-C18 column was used for further analysis. The mobile phase of the binary solvent system was tested with acetonitrile/water and methanol/water, and the ratio of different solvents was tested. Finally, water/acetonitrile containing 0.1% formic acid was selected as the mobile phase, and the ratio of the two was 65:35. Under this condition, kokusaginine had a higher separation degree and a better peak shape from the matrix source.

### 2.3. Method Verification

#### 2.3.1. Specificity

**Figure 1:**
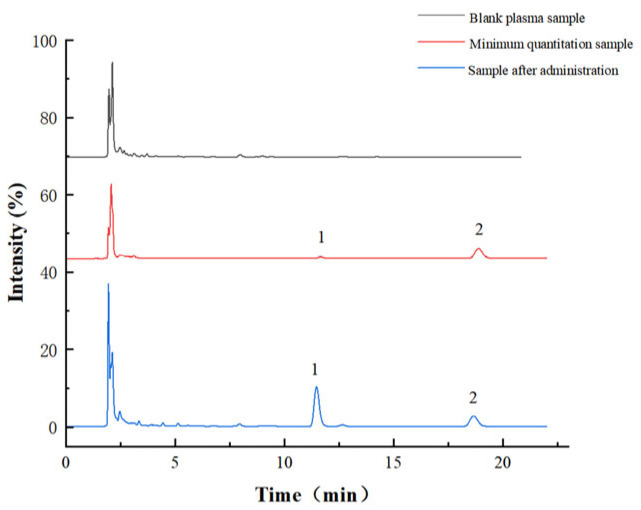
Specialty chromatogram of kokusaginine in rat plasma (1, kokusaginine; 2, dictamine).

**Figure 2:**
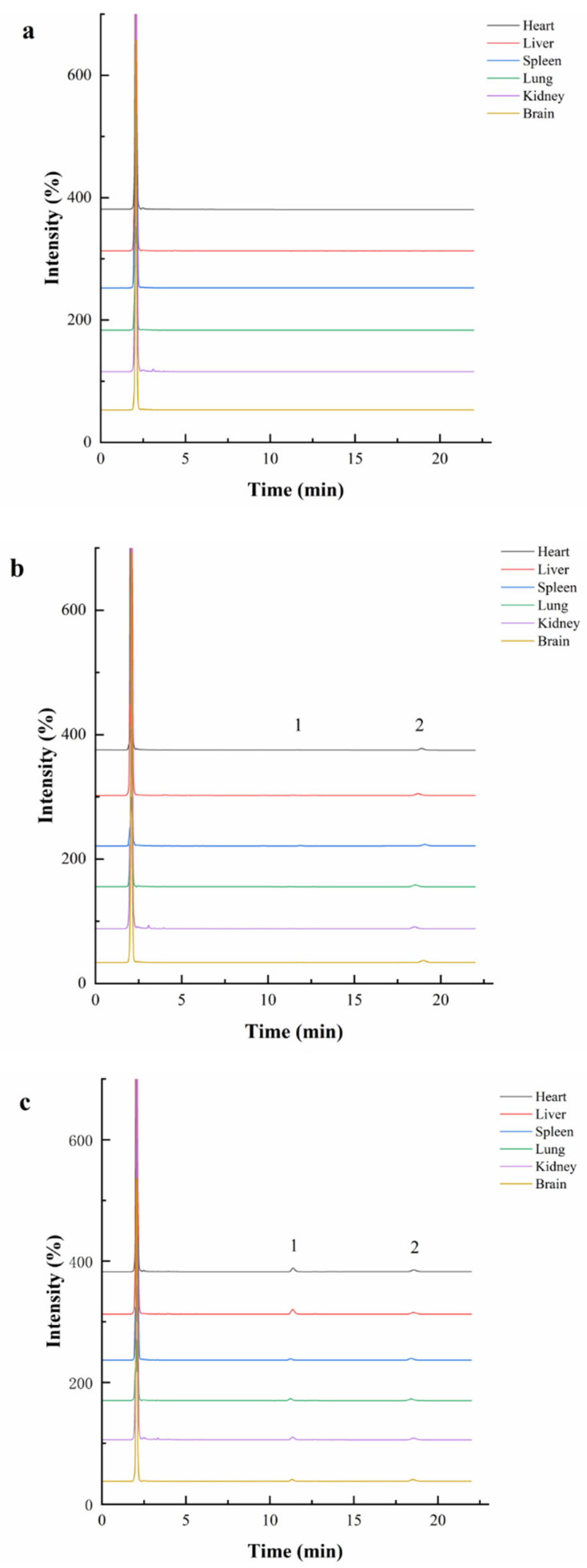
Specialty chromatogram of kokusaginine in the heart, liver, spleen, lung, kidney, and brain of rats. ((**a**), blank sample; (**b**), a blank sample spiked at the LLOQs; (**c**), a sample taken from a rat 120 min after oral administration of 28 mg/kg kokusaginine; 1, kokusaginine; 2, dictamine).

As shown in Figure 1 and Figure 2, no interference peak was observed in the peak region of kokusaginine or dictamine (IS), and there was no interference between the analyte and IS, indicating that the proposed method had high selectivity and specificity.

#### 2.3.2. Standard Curve, Limit of Detection (LOD), and LLOQ

The standard curves, linear ranges, LOD, and LLOQ of the plasma and tissues are shown in Table 1, and all correlation coefficients (r) were above 0.99, indicating that the calibration curves of analytes in various biological substrates were well fitted. The precision (RSD) and accuracy (RE) of the actual concentration at each point on the standard curve were less than 15% of the standard concentration, including the limit of quantitation, in line with the biological analysis standard.

#### 2.3.3. Precision and Accuracy

The analysis results of the intra- and inter-lot accuracy and precision of the QC analyte in rat plasma and tissues at four concentration levels are shown in Table 2. Intra-day accuracy and precision ranged from 1.72 to 11.62% and from 1.65 to 12.57%, respectively. Inter-day accuracy and precision ranged from −7.66 to 11.77% and from −9.4 to 13.49%, respectively. The data showed that the precision and accuracy of the proposed method met the verification requirements and that it was robust and repeatable.

#### 2.3.4. Matrix Effect and Recovery Rate

The results of extraction recovery and the matrix effect in rat plasma and tissues are shown in Table 3. The recoveries of kokusaginine from plasma and tissues ranged from 91.82 to 98.29%, and the RSD values ranged from 0.41 to 4.74%. The matrix effect values ranged from 88.03 to 97.65%, and the RSD ranged from 0.85 to 4.86%. The extraction process of rat plasma was shown to be consistent and repeatable, with negligible effects of the biological matrix on the reaction of the analyte.

#### 2.3.5. Stability

The stability test results of kokusaginine in rat plasma and tissues under different storage and treatment conditions are shown in Table 4. The short-term room-temperature stability (25 °C, 6 h), preparation stability (25 °C, 12 h after sample treatment), long-term frozen stability at −80 °C, and repeated freeze-thaw stability (three freeze-thaw cycles from −20 °C to room temperature) were investigated. The results showed that both RE and RSD values were within the prescribed range (±15%), indicating that kokusaginine did not degrade significantly under the above conditions.

#### 2.3.6. Residual Effect

**Figure 3:**
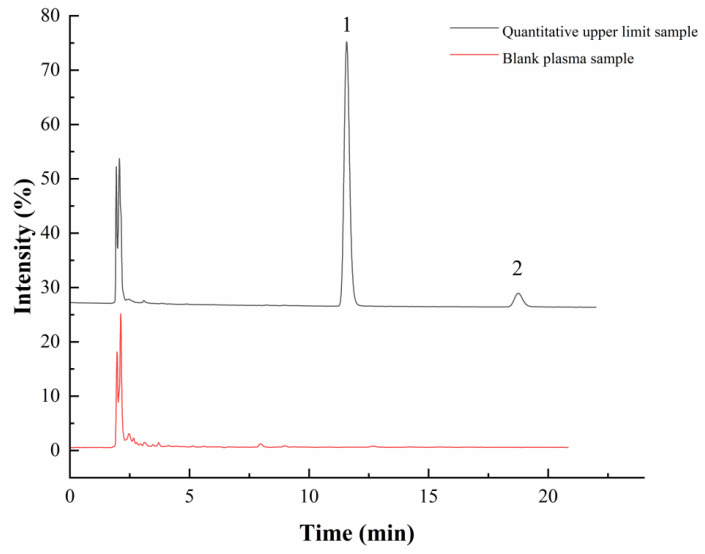
Residual effects of kokusaginine in rat plasma (1, Kokusaginine; 2, Dictamine).

As shown in Figure 3, after the upper limit concentration of the standard curve was injected, it had no effect on the quantification of the double-blank sample.

### 2.4. Metabolic Stability of Rat Liver Microsome

**Figure 4:**
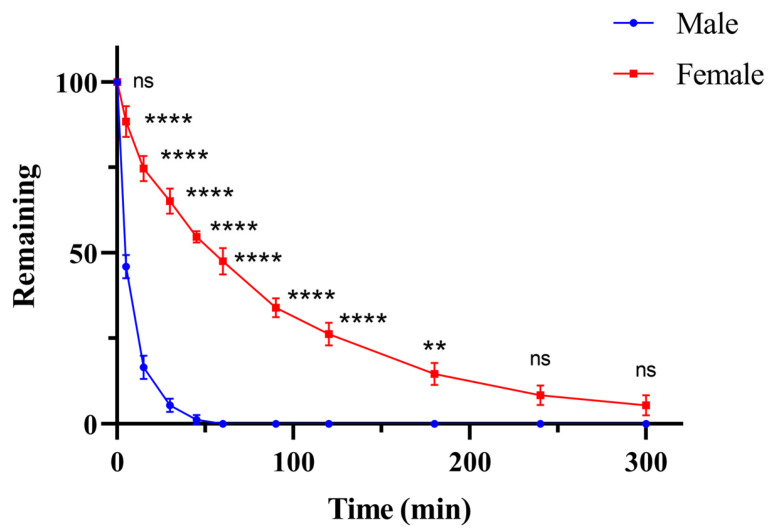
Metabolic elimination curve of kokusaginine in male and female rat liver microsomes (Data are Mean ± SD, *n* = 3, Equal Sex Ratio, ** *p* < 0.01, **** *p* < 0.0001, ns—not significant. compared to the male group).

The metabolic elimination curve of kokusaginine in the liver microsomal incubation system of male and female SD rats is shown in Figure 4, and the remaining percentage is shown in Table 5. Kokusaginine was completely metabolized in the liver microparticles of male SD rats in 60 min and basically metabolized in the liver microparticles of female SD rats in 300 min. The natural logarithm of kokusaginine’s remaining percentage at each time point exhibited a linear regression with incubation time. The linear fitting equations for the liver microsomes of male and female SD rats are y=−0.08935x+4.361 (R^2^ = 0.9557) and y=−0.009938x+4.487 (R^2^ = 0.9603), respectively. The half-lives t_1/2_ of kokusaginine in the liver microsomes of male and female SD rats are 7.76 and 69.73 min, respectively. The CL_int_ of kokusaginine is 89.30 μL/min/mg in the liver microsome of male SD rats and 9.94 μL/min/mg in female SD rats.

### 2.5. Pharmacokinetic Study and Statistical Analysis

**Figure 5:**
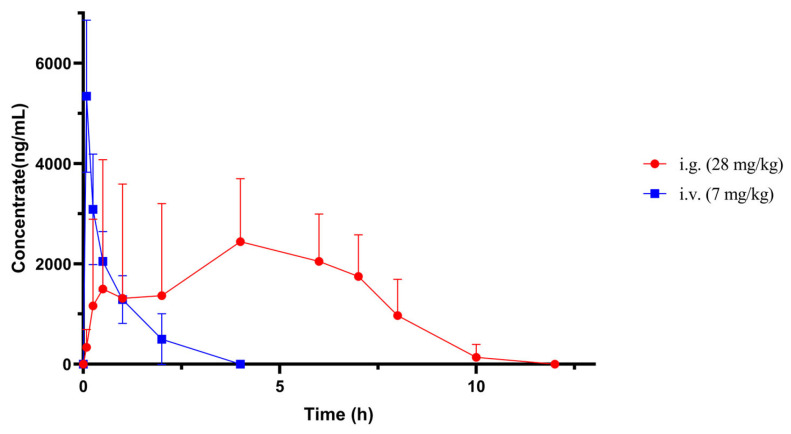
Plasma concentration-time curves of kokusaginine in rats after oral administration of kokusaginine 28 mg/kg and i.v. administration 7 mg/kg (Data are Mean ± SD, *n* = 6, Equal Sex Ratio).

The established method was successfully applied to determine the kokusaginine levels in rat plasma samples for the PK study. The PK parameters of rats (male and female) were obtained via oral (28 mg/kg) and caudal intravenous injections (7 mg/kg) of kokusaginine, as shown in Table 6. Taking the blood collection time point as the horizontal coordinate and the blood drug concentration at each point as the vertical coordinate, the blood drug concentration-time curve of kokusaginine was obtained, as shown in Figure 5. The relevant PK parameters were obtained by PK analysis of the rats according to gender, and the significant differences in each parameter were analyzed, as shown in Table 7. The results showed that the AUMC_(0-∞)_ parameters obtained by oral kokusaginine were significantly different between the sexes (*p* < 0.05).

### 2.6. Tissue Distribution Study

**Figure 6:**
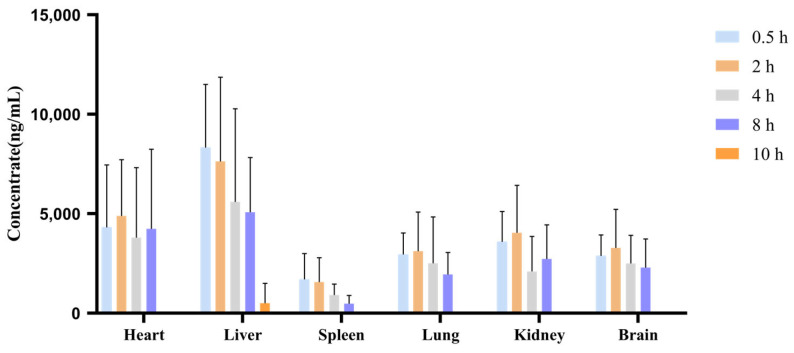
Tissue distribution of kokusaginine in rat tissues after oral administration of 28 mg/kg kokusaginine. (Data are Mean ± SD, *n* = 4, Equal Sex Ratio).

**Figure 7:**
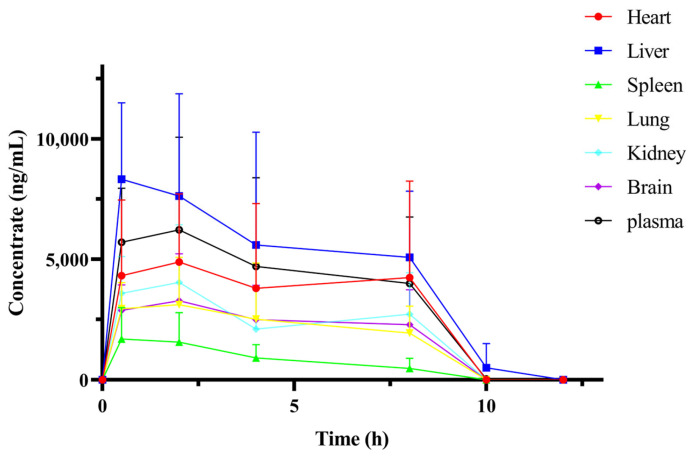
Tissue and plasma concentration-time curves of kokusaginine in rats after oral administration of kokusaginine 28 mg/kg (Data are Mean ± SD, *n* = 4, Equal Sex Ratio).

Figure 6 shows the concentration distribution of kokusaginine in the heart, liver, spleen, lung, kidney, and brain tissues after the oral administration of 28 mg/kg of kokusaginine for 0.5, 2, 4, 8, 10, and 12 h. Figure 7 shows the tissue concentration-time curve of kokusaginine in various tissues and plasma after the oral administration of 28 mg/kg of kokusaginine in rats. The distribution of plasma and tissues and the trend in the PK results are basically consistent. It can be seen from the results that kokusaginine is rapidly and widely distributed throughout the body after 0.5 h of administration, and the average concentration of kokusaginine in various tissues at this time (units of μg/mL) was liver (8.32) > heart (4.32) > kidney (3.59) > lung (2.95) > brain (2.88) > spleen (1.70). After 10 h of oral administration, no tissue was detected except the liver and spleen. The tissue concentration at each time point is shown in Table 8. The tissue-plasma partition coefficient K_p_ calculated by comparing AUC_(tissue)_/AUC_(plasma)_ is shown in Table 9.

The comparison of kokusaginine concentration and pharmacokinetic parameters in different rat tissues at different time points and between different sexes is shown in Table 10. It can be seen from this table that no statistical difference is shown except for gender differences in the pharmacokinetic parameter AUMC_(0-t)_ (*p* < 0.0001) in brain tissues.

## 3. Discussion

The anti-renal fiber effect of kokusaginine was confirmed in our previous study, and other pharmacological effects have also been reported domestically and internationally. However, no current studies have investigated its PKs and tissue distribution. Therefore, to better explore the possible effects of kokusaginine on major organs and its mechanism of action, as well as evaluating its metabolic stability and safety, we studied the PKs, tissue distribution, and bioavailability of kokusaginine in rats, and we conducted metabolic stability experiments combined with cultured rat liver microsomes in vitro. In this study, we used the UPLC method to quantitatively analyze kokusaginine for the first time. Compared to the currently widely used LC-MS method, UPLC provides highly sensitive and high-resolution analysis with lower equipment operating costs and higher sample loads. These characteristics make UPLC particularly suitable for studies requiring many sample analyses while ensuring the accuracy and repeatability of quantitative analyses. The high precision and accuracy of the UPLC method provide us with reliable quantitative data results, and the data processing process is relatively simple.

The PK results of kokusaginine showed that it is rapidly absorbed and rapidly eliminated in rats. The absolute bioavailability of kokusaginine was obtained by oral (28 mg/kg) and intravenous (7 mg/kg) administration. The absolute bioavailability of kokusaginine was about 71.13%, indicating that kokusaginine was absorbed to a significant extent after passing through the digestive tract. The tissue distribution results showed that after a single oral administration of 28 mg/kg of kokusaginine in rats, it was distributed in the heart, liver, spleen, lung, kidney, and brain tissues, among which kokusaginine accumulated most in the liver tissue. Combined with the previous studies on kokusaginine in this subject, the hepatotoxicity of kokusaginine was observed in mice after a single oral administration of 60 mg/kg, suggesting that focus should be placed on the effect of kokusaginine on liver tissue when determining the clinical dosage of kokusaginine. Kokusaginine is also highly enriched in the kidneys, further confirming its role in the treatment of kidney disease. Kokusaginine was almost undetectable 12 h after administration, indicating that it was rapidly eliminated from the tissue without significant retention. In addition, kokusaginine was detected in rat brains, suggesting that it can cross the blood-brain barrier and that it may have the ability to treat brain diseases.

A liver microsomal metabolic stability experiment was conducted to evaluate the metabolism of kokusaginine in the liver microsomes of rats of different genders. In this experiment, the liver intrinsic clearance CL_int_ and half-life t_1/2_ of kokusaginine in the liver microsomes of male SD rats were 89.30 uL/min/mg and 7.76 min, respectively. The intrinsic clearance of CL_int_ and half-life t_1/2_ in the liver of female SD rats were 9.94 μL/min/mg and 69.73 min, respectively, which were significantly different (*p* < 0.05). However, there were no significant differences in the PKs and tissue distribution of rats of different genders, except for the difference in AUMC_(0-∞)_ in plasma (*p* < 0.05) and AUMC_(0-t)_ in brain tissue (*p* < 0.0001), which was statistically significant. The difference between the in vitro and in vivo results can be attributed to several factors. First, the liver microsome experiment had some limitations. Only one batch of liver microsomes was used in this study. However, it is important to evaluate the consistency of microsome activity across batches to control batch-to-batch variability, and we did not evaluate analytes for non-sex-specific metabolism, to ensure robustness of the results. Second, in vitro liver microsomal experiments reflect only the liver metabolism and do not take into account other metabolic pathways or systemic effects that may occur in vivo. In addition, the dosing settings may affect the pharmacokinetics and tissue distribution of kokusaginine, which cannot be captured in simple microsomal trials. Finally, significant AUMC values in plasma and brain tissue suggest that kokusaginine may experience different metabolic fates in these areas, which may be influenced by sex-specific physiological or biochemical processes. Further research is needed to elucidate the multiple metabolic pathways of kokusaginine in the body and to understand how these pathways are affected by sex.

In summary, this study established and validated a new method for the determination of kokusaginine via UPLC and studied the PKs, tissue distribution, and bioavailability of kokusaginine in rats, as well as the metabolic stability of rat liver microsomes incubated in vitro. It provides a reliable and economical method for the determination of kokusaginine in various biological substrates. The absolute oral bioavailability of kokusaginine is good, and after oral administration, the drug is rapidly absorbed in the blood of rats and widely distributed throughout the body. Tissue distribution experiments also demonstrated that kokusaginine was absorbed in multiple tissues, suggesting that kokusaginine had many potential target organs. The results of this study provide reliable data support for a comprehensive understanding of the PKs of kokusaginine.

## 4. Materials and Methods

### 4.1. Materials

#### 4.1.1. Chemicals and Reagents

**Figure 8:**
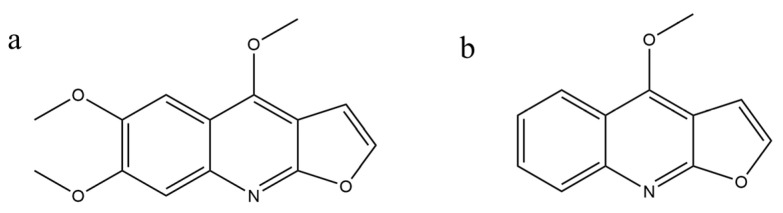
Chemical structures of kokusaginine (**a**) and dictamine (**b**).

Kokusaginine was synthesized in the laboratory with a purity of >99%. The internal standard dictamine (Batch number: 111654-200301) was purchased from the China National Institute for Food and Drug Control (Beijing, China), and the chemical structures of the two are shown in Figure 8. Acetonitrile and formic acid were purchased from Anaqua Chemicals Supply (Shanghai, China) Co., Ltd., and methanol was bought from Anhui Tedia High Purity Solvents Co., Ltd. (Anqing, China); the solvents were HPLC grade. Sodium carboxymethyl cellulose salt (purity: Laboratory Reagent, LR) was purchased from Shanghai Shanpu Chemical (Shanghai, China) Co., Ltd. The ultrapure water came from China Resources Ebao Beverage (Changsha, China) Co., Ltd. A phase I metabolic stability kit was purchased from Huizhi and Source Biotechnology (Suzhou, China) Co., Ltd., including liver microsomes from male rats (Sprague Dawley), catalog number 0111D1.01, and liver microsomes from female rats (Sprague Dawley), catalog number 0111D1.02.

#### 4.1.2. Animals

Half-male SD rats weighing 200–220 g were purchased from Hunan Slack Jingda Co., Ltd. (Changsha, China). Before the experiment, all rats were divided into male and female groups, placed in plastic cages, and adapted to the conditions of constant temperature (23 ± 2 °C), humidity (55 ± 5%), and a daily light and dark period of 12 h for 1 week, during which the rats could eat and drink freely. In addition, the mice fasted for 12 h before administration with unrestricted water intake. All animal experimental procedures were carried out in accordance with national guidelines, and the animal experimental research program was approved by the Xiangya Hospital of Central South University Animal Ethics Committee, ethics approval number: 2023111766.

#### 4.1.3. Instrumentation and Chromatographic Conditions

UPLC was used to determine the contents of kokusaginine and internal standard dictamine in biological samples. The chromatography was performed on an Agilent ZORBAX SB-C18 column (4.6 × 250 mm, 5 μm) by an Agilent 1290 Infinity II UHPLC (Agilent Technologies Co., Ltd., Ltd., California, United States) system for the chromatographic separation of kokusaginine and IS. The samples were eluted with 0.1% formic acid aqueous solution (A) and acetonitrile (B) at a constant column temperature of 25 °C, a flow rate of 1.0 mL/min, a detection wavelength of 250 nm, and a sample size of 10 μL. The total time required for the analysis of a single sample was 22 min.

#### 4.1.4. Preparation of Stock Solution and Working Solution

A certain amount of kokusaginine was dissolved in methanol to prepare a kokusaginine reserve solution with a final concentration of 1 mg/mL. The kokusaginine reserve solution was diluted with methanol to prepare the kokusaginine calibration solution with the final concentrations of 240, 200, 150, 100, 50, 10, 5, 1, and 0.5 μg/mL, respectively. The internal standard solution was dissolved into 528 μg/mL of internal standard reserve solution by methanol and further diluted into 52.8 μg/mL of internal standard working solution by methanol. Kokusaginine and the internal standard dictamine reserve solution were stored at −20 °C, and the working solution was stored at 4 °C.

#### 4.1.5. Preparation of Calibration Standard and Quality Control (QC) Samples

Blank plasma or tissues were mixed with the working solution to prepare calibration standards and QC samples. Amounts of 10 μL of kokusaginine working solution and 10 μL of IS solution were added to 100 μL of blank rat plasma or tissue samples to obtain calibration standard samples. The concentration of the calibration standard was the same: 12,000, 10,000, 5000, 2500, 1000, 500, 250, 100, 50, and 25 ng/mL.

The final concentrations of kokusaginine in the QC rat plasma were 25 ng/mL (Lower Limit of Quantification, LLOQ), 50 ng/mL (Low QC, LQC), 2.5 μg/mL (Medium QC, MQC), and 7.5 μg/mL (High QC, HQC). The final concentrations of kokusaginine in the liver QC were 50 ng/mL (LLOQ), 100 ng/mL (LQC), 2.5 μg/mL (MQC), and 12 μg/mL (HQC). The final concentrations of kokusaginine in the QC of the remaining tissues were 50 ng/mL (LLOQ), 100 ng/mL (LQC), 2.5 μg/mL (MQC), and 7.5 μg/mL (HQC), and all of these solutions were stored at 4 °C. To verify the presence of interference, a zero sample (no analyte, but with IS) and a blank sample (no analyte, no IS) were included in each analytical batch. The calibrators and QC samples were freshly made on the same day the sample analysis took place to ensure accuracy and were pre-treated according to the same procedures as the study samples, as described in Section 4.1.6.

#### 4.1.6. Sample Preparation

The samples were prepared by protein precipitation and liquid-liquid extraction. There were 100 μL of each biological sample and 10 μL of internal standard solution. The mixture was swirled (XW-80A, Shanghai Qingpu Huxi Instrument Factory, Shanghai, China) for 1 min and then swirled with 600 μL of acetonitrile for 1 min, and the mixture was centrifuged at 14,500 r·min^−1^ (Dalong Xingchuang Experimental Instrument (Beijing) Co., Ltd., D-2012plus, Beijing, China) for 10 min. Then, all supernatants were collected, and 1 mL of ethyl acetate was added, swirled for 1 min, and centrifuged at 14,500 r·min^−1^ for 10 min. Finally, the nitrogen blower (Shaying Scientific Instrument (Shanghai) Co., Ltd., HGC-24-026, Shanghai, China) was used to dry at 35 °C. The dried substance was dissolved in 200 μL of acetonitrile and 0.1% formic acid water (35/65, *v*/*v*), swirled for 1 min, centrifuged for 10 min at 14,500 r·min^−1^, and injected with 10 μL of supernatant into a UPLC system, and the chromatographic peak area was recorded. The tissue samples were ground to a tissue homogenate with 0.9% saline (1:3, *w*/*v*), and the individual tissue samples were treated using the same procedure as the plasma samples.

#### 4.1.7. Solution Preparation

An amount of 2.8 mg/mL of kokusaginine oral solution was prepared by dissolving kokusaginine in 0.5% CMC-Na solution. Kokusaginine was dissolved in a mixed solution system (10% DMSO, 70% PEG, and 20% normal saline) to prepare 0.7 mg/mL of kokusaginine injection. Kokusaginine was dissolved in methanol, and a solution of 5 mM was prepared for the liver microsomal stability test.

### 4.2. Methods

#### 4.2.1. Methodological Validation

The study method was validated in accordance with the Guidelines for the Validation of Bioanalytical Methods for Industry issued by the US Food and Drug Administration (US-FDA, 2018) and the Chinese Pharmacopoeia Commission (2020). The specificity, linearity, precision, accuracy, matrix effect, recovery rate, stability, and residual effect of the method were verified under different conditions.

#### 4.2.2. Specificity

The specificity of the method was assessed by analyzing different biological samples of plasma and various tissues. Specificity was assessed in blank substrates, blank substrates with kokusaginine (LLOQ) and IS working solutions, and rat biological samples after the oral administration of kokusaginine. The method is considered selective if the response of the interference component does not exceed 20% of the response of the LLOQ analyte and 5% of the IS response in the LLOQ sample during the retention time.

#### 4.2.3. Standard Curve, Limit of Detection (LOD), and LLOQ

Calibration standard samples (*n* = 9) of rat plasma and tissues were taken each was measured 3 times, and the average value was taken. A standard curve was drawn using the ratio of the calculated analyte to the peak area of the corresponding IS (Y) and analyte concentration (X), and the correlation coefficient (r) should be greater than 0.99. The Limit of Detection (LOD) was estimated using analysis software when the SNR was 3, and the LLOQ when the SNR was 10 was used as the lowest concentration of the calibration curve. The accuracy of the inverse concentration of the calibration standard should be within ±15% of the nominal value, and the limit of quantitation should be within ±20%.

#### 4.2.4. Precision and Accuracy

Precision (relative standard deviation, RSD) and accuracy (relative error, RE) within and between batches were examined by analyzing QC samples for three consecutive batches with six replicates per batch at four concentration levels (L, M, HQC, and LLOQ). In addition to the LLOQ, the QC sample should be within ±20% of the nominal value, and the average concentration of each QC level in the lot and between the lot RE should be within ±15% of the nominal value. In addition to the LLOQ, the QC sample should be within 20% inside and out, and the intra- and inter-lot RSD for each QC level measured should be within 15%.

#### 4.2.5. Matrix Effect and Recovery Rate

After extracting the blank matrix from 6 different rats and then adding kokusaginine (L, M, and HQC) and an internal standard, the peak area was measured, and the matrix effect was evaluated and compared with the peak area of kokusaginine and the internal target pure solution (methanol). The extraction recovery was evaluated and compared with six batches of QC samples with three levels of concentration (L, M, and HQC). The acceptance criteria were a difference in QC concentration and recovery of ≤30% and a coefficient of variation of the matrix effect of ≤15%.

#### 4.2.6. Stability

The stability of QC samples with three concentrations (L, M, and HQC) was evaluated by 6 tests under different conditions, including the (1) short-term stability at room temperature (25 °C) for 6 h; the (2) preparation stability at 24 h under an automatic sampler (25 °C); the (3) three freeze-thaw cycles at −20 °C; and the (4) long-term stability at −80 °C for 30 days. The acceptance criterion was that the deviation between the mean value of each concentration and the standard concentration should be within the range of ±15%.

#### 4.2.7. Residual Effect

To verify the residues, a blank sample (no analyte, no IS) was analyzed after analyzing the upper concentration sample of the standard curve. In the event that a peak IS was detected in a double-blank sample after the upper limit concentration of the standard curve for the same retention time as the analyte and IS, the response of that peak should be no greater than 20% of the lower limit of quantitation (LLOQ) and no greater than 5% of IS.

#### 4.2.8. Metabolic Stability of Rat Liver Microsome

Amounts of 531 μL of PBS buffer solution, 30 μL of rat liver microsomes of different genders (20 mg/mL, final concentration of 1 mg/mL), and 3 μL of kokusaginine (5 mM, final concentration of 0.025 mM) were added into the vortex and mixed evenly. After pre-incubation at 37 °C for 5 min, 36 μL of the NADPH regeneration system was added to start the reaction. After incubation at 37 °C for 0, 5, 15, 30, 45, 60, 90, 120, 180, 240, and 300 min, 50 μL was added to 50 μL of cold acetonitrile (−20 °C) containing internal standard dictamine solution (528 μg/mL; final concentration of 3.3 μg/mL), terminating the reaction. After centrifugation at 14,500 r·min^−1^ for 10 min, the supernatant was added to the sample, and the remaining percentage and related parameters were calculated.

The remaining percentage was the ratio between the concentration of kokusaginine at each time point and the mass concentration at incubation for 0 min. The relevant formula is shown in Equation (1):

(1) $$Residual\;percentage(\%)=(\frac{C(t)}{C(0)})\;\times \;100\%$$

where C(t) is the concentration value of the analyte at incubation time t, and C(0) is the concentration value of the analyte at incubation time 0.

Linear regression was performed on the natural logarithm of the drug residue percentage and incubation time at each time point to obtain the slope k, and then the half-life was calculated by the following formula:

(2) $$t_{1/2}=-\frac{In(2)}{k}$$

where ln(2) is the logarithm of 2 based on the natural logarithm, approximately equal to 0.693, and k is the rate constant of drug metabolism.

Finally, the half-life of the drug is related to its clearance rate. By measuring the half-life of the drug in the liver microsomal incubation system, the internal clearance rate of the drug can be estimated. Equation (3) was used to calculate the internal clearance CL_int_ of kokusaginine [21]:

(3) $${CL}_{int(\mu L/min/mg)}=\frac{In(2)\times Incubation\;volume(\mu L)}{t_{1/2}(min)\times Liver\;microsome\;mass\;(mg)}$$

where CL_int_ is the internal clearance rate (μL/min/mg), indicating the volume of liver microsomes that can be cleared per unit mass in unit time. In(2) is the logarithm of 2 based on the natural logarithm, approximately equal to 0.693. Incubation volume (mL) is the total volume of liver microsomes or liver cells used in the experiment. Liver microsome mass (mg) is the mass of the liver microsome used in the experiment.

#### 4.2.9. Pharmacokinetic Study

SD rats (*n* = 12; 6 males and 6 females; weight 200 ± 10 g) were randomly divided into 2 groups (male and female half). After the experiment, all the rats were rapidly anesthetized with isoflurane and immediately killed by cervical dislocation. Rats in the first group were given an oral solution of 2 mL of kokusaginine (2.8 mg/mL). Blood samples of 300 μL were collected from the orbital venous plexus before administration, 0, 5, 15, and 30 min, and 1, 2, 4, 6, 7, 8, 10, 12, and 24 h after administration. After centrifugation, the supernatant was taken and placed in an EP tube, frozen, and stored at −80 °C for analysis. Rats in the second group were injected intravenously, each rat was injected with 2 mL of kokusaginine (0.7 mg/mL) through the tail vein, and 300 μL of blood was collected from the orbital venous plexus before administration, 0, 5, 15, and 30 min, and 1, 2, 4, 8, 10, and 12 h after administration. After centrifugation, the supernatant was taken and placed in an EP tube, frozen, and stored at −80 °C for analysis.

#### 4.2.10. Tissue Distribution Study

SD rats (*n* = 28; 14 males and 14 females; weight 200 ± 10 g) were randomly divided into 7 groups of 4 (half male and half female). One group was sacrificed directly as a blank group, and the other five groups were sacrificed 0.5, 2, 4, 8, 10, and 12 h after the oral administration of 2 mL of kokusaginine (2.8 mg/mL). The method of sacrifice was cervical dislocation. The aorta was then perfused with saline until the blood was completely removed, and the heart, liver, kidneys, spleen, lungs, brain, and plasma were removed. The residual blood on the surface was rinsed with normal saline, dried with filter paper, and weighed. Then, it was ground evenly with triploid saline and frozen at −80 °C.

#### 4.2.11. Pharmacokinetics and Statistical Analysis

The PK parameters were calculated in WinNonlin Phoenix 8.3.5 (v6.4, Pharsight, Mountain View, CA, USA) and analyzed using the non-atrioventricular model. The main PK parameters and corresponding definitions are shown in Table S1. Absolute bioavailability was calculated as AUClast in the PO administration group divided by AUClast in the IV administration group as follows [22]:

(4) $$Absolute\;bioavailability(\%)=\frac{{AUC}_{PO}}{{AUC}_{IV}}\times \frac{{Dose}_{IV}}{{Dose}_{PO}}\times 100\%$$

Drugs reach dynamic equilibrium in tissue and plasma over a certain period of time, and AUC(tissue)/AUC(plasma) can reflect the distribution ratio of drugs between tissue and plasma, known as the tissue-plasma distribution coefficient (Kp) [23]:

(5) $$Tissue–plasma\;partition\;coefficients\;(Kp)=\frac{{AUC}_{tissue}}{{AUC}_{plasma}}$$

GraphPad software (GraphPad Prism 8.0.2; GraphPad Software, Inc., La Jolla, CA, USA) was used to perform statistical analysis. *p* < 0.05 was considered statistically significant, and the data are represented by the mean ± standard deviation (SD) and relative standard deviation (RSD).

## 5. Conclusions

In summary, this study established and validated a new UPLC method for the determination of kokusaginine, focusing on the evaluation of its PKs, tissue distribution, and bioavailability in rats, and a comparative analysis of males and females was conducted. This method requires relatively low-cost reagents, consumables, and equipment and has a short analysis time and high repeatability. It can be considered that this method provides a reliable and economical method for the determination of kokusaginine in various biological substrates. Our findings suggest that the absolute oral bioavailability of kokusaginine is high in both sexes; it can be rapidly absorbed into the bloodstream and is widely distributed throughout the body. Tissue distribution experiments showed that kokusaginine can be absorbed in a variety of tissues, suggesting that the drug has multiple potential target organs. Notably, significant sex differences were shown in metabolic stability, assessed in rat liver microsomes, rat plasma AUMC_(0-∞)_, and brain tissue AUMC_(0-t)_, highlighting the importance of considering sex in drug metabolism studies. A comprehensive evaluation of kokusaginine’s PKs, including its oral bioavailability and tissue distribution, provides valuable insights into its potential therapeutic applications. The findings of this study provide a solid data basis for developing gender-specific drug administration strategies and contribute to a nuanced understanding of the PK behavior of kokusaginine in a clinical context.

## Figures and Tables

**Table 1:** Linearity regression functions of kokusaginine in different rat matrices (*n* = 3).

Biological Sample	Calibration Curve *	Linear Range	R^2^	LOD	LLOQ
Plasma	y=0.001x−0.003	25 ng/mL–10 μg/mL	0.999	20 ng/mL	25 ng/mL
Heart	y=0.001x−0.008	50 ng/mL–10 μg/mL	0.998	25 ng/mL	50 ng/mL
Liver	y=0.001x−0.022	50 ng/mL–10 μg/mL	0.992	25 ng/mL	50 ng/mL
Spleen	y=0.001x+0.109	50 ng/mL–10 μg/mL	0.996	25 ng/mL	50 ng/mL
Lung	y=0.0018x−0.003	50 ng/mL–10 μg/mL	0.995	25 ng/mL	50 ng/mL
Kidney	y=0.001x−0.004	50 ng/mL–10 μg/mL	0.991	25 ng/mL	50 ng/mL
Brain	y=0.001x−0.003	50 ng/mL–10 μg/mL	0.992	25 ng/mL	50 ng/mL

* In linear regression equations, y is the peak-area ratio of analytes to IS and x (ng/mL) is the plasma concentration of analyte.

**Table 2:** Accuracy and precision of kokusaginine in rat plasma and heart, liver, spleen, lung, kidney, and brain (six times a day for three days; Data are Mean ± SD, *n* = 6).

Matrix	Sample	Nominal Concentration (ng/mL)	Intra-Day			Inter-Day		
			Measured Concentration (Mean ± SD)	Precision (% RSD)	Accuracy (% Bias)	Measured Concentration (Mean ± SD)	Precision (% RSD)	Accuracy (% Bias)
Plasma	LLOQ	25	23.31 ± 2.30	9.85	−6.78	22.6 5± 2.85	12.57	−9.4
	LQC	50	48.26 ± 5.61	11.62	−3.48	46.93 ± 5.22	11.12	−6.14
	MQC	2500	2679.68 ± 145.81	5.44	7.19	2649.59 ± 174.79	6.60	5.98
	HQC	7500	7708.11 ± 369.50	4.79	2.77	7753.25 ± 485.84	6.27	3.38
Heart	LLOQ	50	49.86 ± 4.02	8.06	−0.28	49.10 ± 4.42	9.00	−1.79
	LQC	100	94.65 ± 6.15	6.50	−5.35	96.90 ± 11.14	11.5	−3.10
	MQC	2500	2376.07 ± 85.59	3.60	−4.96	2385.06 ± 94.09	3.94	−4.60
	HQC	7500	7450.41 ± 128.11	1.72	−0.66	7415.68 ± 133.06	1.79	−1.12
Liver	LLOQ	50	49.28 ± 5.61	11.39	−1.44	49.49 ± 5.65	11.42	−1.02
	LQC	100	92.34 ± 8.05	8.72	−7.66	90.95 ± 7.04	7.74	−9.05
	MQC	2500	2330.29 ± 41.09	1.76	−6.79	2336.38 ± 38.65	1.65	−6.54
	HQC	12,000	12,704.28 ± 236.13	1.86	5.87	12,772.42 ± 287.27	2.25	6.44
Spleen	LLOQ	50	55.89 ± 4.66	8.33	11.77	56.74 ± 4.21	7.43	13.49
	LQC	100	110.66 ± 3.90	3.52	10.66	109.46 ± 4.81	4.39	9.46
	MQC	2500	2490.36 ± 156.59	6.29	−0.39	2498.07 ± 189.88	7.6	−0.08
	HQC	7500	7231.74 ± 323.19	4.47	−3.58	7337.51 ± 354.71	4.83	−2.17
Lung	LLOQ	50	55.32 ± 4.81	8.69	10.63	54.72 ± 4.68	8.55	9.43
	LQC	100	102.80 ± 4.31	4.19	2.80	102.92 ± 5.13	4.99	2.92
	MQC	2500	2556.36 ± 153.11	5.99	2.25	2518.71 ± 138.42	5.5	0.75
	HQC	7500	7780.28 ± 310.83	4.00	3.74	7775.57 ± 276.55	3.56	3.67
Kidney	LLOQ	50	49.71 ± 5.24	10.54	−0.57	50.09 ± 4.49	8.95	0.18
	LQC	100	99.68 ± 6.37	6.39	−0.32	99.51 ± 5.51	5.53	−0.49
	MQC	2500	2701.02 ± 186.34	6.90	8.04	2621.29 ± 253.94	9.69	4.85
	HQC	7500	8327.29 ± 275.44	3.31	11.03	8267.69 ± 303.08	3.67	10.24
Brain	LLOQ	50	53.68 ± 2.62	4.89	7.37	52.44 ± 2.90	5.52	4.88
	LQC	100	100.90 ± 3.55	3.52	0.90	101.03 ± 4.36	4.32	1.03
	MQC	2500	2397.19 ± 136.22	5.68	−4.11	2396.62 ± 119.93	5.00	−4.14
	HQC	7500	7423.29 ± 174.59	2.35	−1.02	7417.00 ± 161.68	2.18	−1.11

**Table 3:** Extraction recovery and matrix effects of kokusaginine in rat plasma and in the heart, liver, spleen, lung, kidney, and brain (Data are Mean ± SD, *n* = 6).

Matrix	Sample	Nominal Concentration (ng/mL)	Extraction Recovery		Matrix Effect	
			Mean ± SD (%)	RSD (%)	Mean ± SD (%)	RSD (%)
Plasma	LQC	50	97.12 ± 4.60	4.74	90.56 ± 2.45	2.70
	MQC	2500	98.06 ± 3.45	3.52	97.65 ± 3.20	3.28
	HQC	7500	96.89 ± 3.17	3.27	95.47 ± 1.67	1.74
Heart	LQC	100	95.17 ± 2.00	2.10	95.45 ± 4.64	4.86
	MQC	2500	91.82 ± 2.10	2.28	95.62 ± 2.31	2.42
	HQC	7500	95.37 ± 2.35	2.46	93.22 ± 1.88	2.01
Liver	LQC	100	94.59 ± 1.43	1.52	96.98 ± 1.24	1.28
	MQC	2500	93.17 ± 1.04	1.12	91.61 ± 2.88	3.14
	HQC	12,000	92.52 ± 1.31	1.42	91.78 ± 1.35	1.47
Spleen	LQC	100	96.31 ± 1.48	1.54	95.57 ± 1.86	1.95
	MQC	2500	96.72 ± 3.03	3.14	88.03 ± 3.76	4.27
	HQC	7500	97.81 ± 1.92	1.96	90.37 ± 1.98	2.19
Lung	LQC	100	93.77 ± 1.67	1.78	94.01 ± 1.88	2.00
	MQC	2500	98.29 ± 1.69	1.72	88.96 ± 2.43	2.73
	HQC	7500	96.99 ± 0.40	0.41	92.10 ± 3.93	4.26
Kidney	LQC	100	93.60 ± 1.34	1.44	93.48 ± 2.19	2.34
	MQC	2500	94.89 ± 2.68	2.82	92.46 ± 2.84	3.07
	HQC	7500	96.45 ± 1.34	1.39	94.37 ± 0.99	1.04
Brain	LQC	100	93.84 ± 3.77	4.02	90.19 ± 1.57	1.74
	MQC	2500	95.27 ± 1.95	2.05	94.64 ± 3.21	3.39
	HQC	7500	93.64 ± 2.34	2.49	95.29 ± 0.81	0.85

**Table 4:** Stability of kokusaginine in plasma, heart, liver, spleen, lung, kidney, and brain of rats under different conditions (Data are Mean ± SD, *n* = 6).

Matrix	Sample	Nominal Concentration (ng/mL)	Room Temperature (25 °C, 6 h)			Autosampler (25 °C,12 h)			Freeze Thaw (−20 °C, 3 Cycles)			Long Term (−80 °C, 30 d)		
			Measured Concentration (Mean ± SD)	RSD (%)	Bias (%)	Measured Concentration (Mean ± SD)	RSD (%)	Bias (%)	Measured Concentration (Mean ± SD)	RSD (%)	Bias (%)	Measured Concentration (Mean ± SD)	RSD (%)	Bias (%)
Plasma	LQC	50	43.78 ± 2.15	4.91	−12.44	47.63 ± 2.92	6.13	−4.73	49.17 ± 5.72	11.62	−1.66	44.39 ± 2.12	4.78	−11.21
	MQC	2500	2261.59 ± 80.56	3.56	−9.54	2548.07 ± 88.90	3.49	1.92	2445.90 ± 88.40	3.61	−2.16	2384.94 ± 127.51	5.35	−4.60
	HQC	7500	7392.81 ± 112.43	1.52	−1.43	7551.45 ± 239.69	3.17	0.69	7832.13 ± 354.98	4.53	4.43	7090.55 ± 171.13	2.41	−5.46
Heart	LQC	100	92.26 ± 6.00	6.50	−7.74	89.67 ± 2.25	2.51	−10.33	96.93 ± 5.46	5.64	−3.07	88.55 ± 3.92	4.42	−11.45
	MQC	2500	2386.34 ± 29.10	1.22	4.55	2407.78 ± 93.14	3.87	−3.69	2421.63 ± 122.73	5.07	−3.13	2443.30 ± 48.31	1.98	−2.27
	HQC	7500	7587.53 ± 135.08	1.78	1.17	7543.89 ± 140.00	1.86	0.59	7521.79 ± 136.67	1.82	0.29	7269.29 ± 74.40	1.02	−3.08
Liver	LQC	100	89.41 ± 3.15	3.52	−10.59	100.89 ± 5.07	5.02	0.89	102.02 ± 5.93	5.81	2.02	109.55 ± 1.42	1.29	9.55
	MQC	2500	2325.02 ± 37.69	1.62	−7.00	2348.72 ± 50.19	2.14	−6.05	2331.13 ± 24.95	1.07	−6.75	2281.87 ± 41.10	1.80	−8.73
	HQC	12,000	12,654.33 ± 316.31	2.5	5.45	13,051.09 ± 200.50	1.54	8.76	12,564.12 ± 455.05	3.62	4.7	12,987.20 ± 213.01	1.64	8.23
Spleen	LQC	100	104.39 ± 2.55	2.44	4.39	104.17 ± 3.89	3.73	4.17	105.77 ± 4.52	4.27	5.77	105.21 ± 5.40	5.14	5.21
	MQC	2500	2413.72 ± 165.51	6.86	−3.45	2550.39 ± 167.96	6.59	2.02	2426.46 ± 208.62	8.6	−2.94	2587.44 ± 182.66	7.06	3.50
	HQC	7500	7218.87 ± 325.84	4.51	−3.75	7813.87 ± 92.45	1.18	4.18	7687.45 ± 207.98	2.71	2.5	7638.03 ± 140.61	1.84	1.84
Lung	LQC	100	97.28 ± 5.94	6.11	−2.72	98.21 ± 3.67	3.74	−1.79	99.23 ± 4.71	4.71	−0.77	102.32 ± 2.29	2.23	2.32
	MQC	2500	2460.03 ± 65.89	2.68	−1.6	2583.60 ± 209.23	8.10	3.34	2562.40 ± 170.65	6.66	2.5	2555.32 ± 257.59	10.08	2.21
	HQC	7500	7667.54 ± 180.75	2.36	2.23	7754.81 ± 284.20	3.66	3.40	7773.35 ± 278.80	3.59	3.64	7870.55 ± 171.68	2.18	4.94
Kidney	LQC	100	95.70 ± 6.08	6.35	−4.30	97.65 ± 4.40	4.51	−2.35	94.20 ± 1.69	1.8	−5.8	92.56 ± 3.66	3.95	−7.44
	MQC	2500	2553.66 ± 110.90	4.34	2.15	2580.51 ± 227.08	8.80	3.22	2659.99 ± 119.13	4.48	6.40	2517.20 ± 138.14	5.49	0.69
	HQC	7500	7674.21 ± 523.54	6.82	2.32	7897.68 ± 145.69	1.84	5.3	7643.59 ± 516.77	6.76	1.91	7795.67 ± 97.40	1.25	3.94
Brain	LQC	100	101.50 ± 3.20	3.15	1.5	103.48 ± 2.71	2.62	3.48	97.14 ± 4.63	4.77	−2.86	100.06 ± 4.62	4.62	0.06
	MQC	2500	2430.31 ± 40.20	1.65	−2.79	2439.79 ± 88.26	3.62	−2.41	2368.88 ± 154.10	6.51	−5.25	2334.77 ± 73.41	3.14	−6.61
	HQC	7500	7533.63 ± 222.77	2.96	0.45	7459.93 ± 186.59	2.50	−0.53	7584.65 ± 315.76	4.16	1.13	7643.92 ± 197.84	2.59	1.92

**Table 5:** Residual percentage of kokusaginine in liver microsomes of male and female SD rats (Data are Mean ± SD, *n* = 3, Equal Sex Ratio).

Time (min)	Remaining Area %	
	Male Rat Liver Microsomes	Female Rat Liver Microsomes
0	100.00 ± 0.00	100.00 ± 0.00
5	45.95 ± 3.78	88.40 ± 4.52
15	16.51 ± 3.42	74.68 ± 3.71
30	5.42 ± 2.00	65.11 ± 3.68
45	1.87 ± 1.22	54.71 ± 1.70
60	-	47.56 ± 3.84
90	-	33.95 ± 2.79
120	-	26.30 ± 3.35
180	-	14.62 ± 3.21
240	-	8.40 ± 2.81
300	-	5.43 ± 2.98

**Table 6:** Pharmacokinetic parameters of kokusaginine in rats following oral (28 mg/kg) and intravenous administration (7 mg/kg) (Data are Mean ± SD, *n* = 6, Equal Sex Ratio).

Pharmacokinetic Parameters	Unit	i.g. (28 mg/kg)	i.v. (7 mg/kg)
T_1/2_	h	0.69 ± 0.43	0.69 ± 0.38
T_max_	h	3.79 ± 2.76	-
C_max_	μg/mL	2.97 ± 1.94	5.34 ± 1.52
Vz	L/kg	1.79 ± 1.12	1.76 ± 0.24
CL	L/h/kg	1.59 ± 1.05	2.11 ± 0.88
AUC_(0-t)_	h·μg/mL	15.30 ± 8.47	3.80 ± 1.71
AUC_(0-∞)_	h·μg/mL	86.22 ± 10.15	4.02 ± 2.26
AUMC_(0-t)_	h·h·μg/mL	62.55 ± 39.36	2.07 ± 1.03
AUMC_(0-∞)_	h·h·μg/mL	66.36 ± 43.55	4.69 ± 5.28
MRT_(0-t)_	h	4.69 ± 1.10	0.61 ± 0.07
MRT_(0-∞)_	h	4.54 ± 1.23	0.95 ± 0.48
Bioavailability	%	71.13 ± 12.75	

**Table 7:** Comparative pharmacokinetic parameters of oral (28 mg/kg) and intravenous administration (7 mg/kg) in rats (Data are Mean ± SD, *n* = 6, Equal Sex Ratio).

Pharmacokinetic Parameters	Unit	i.g. (28 mg/kg)			i.v. (7 mg/kg)		
		Male Rats	Female Rats	*p* Value	Male Rats	Female Rats	*p* Value
T_1/2_	h	0.88 ± 0.41	0.50 ± 0.09	>0.9999	0.54 ± 0.18	0.92 ± 0.58	>0.9999
T_max_	h	6.00 ± 0.00	1.58 ± 2.10	>0.9999	-	-	-
C_max_	μg/mL	2.67 ± 0.71	3.27 ± 2.94	>0.9999	4.89 ± 1.43	6.02 ± 1.89	>0.9999
Vz	L/kg	2.36 ± 0.71	1.22 ± 1.29	>0.9999	1.83 ± 0.29	1.64 ± 0.08	>0.9999
Cl	L/h/kg	2.00 ± 0.72	1.18 ± 1.32	>0.9999	2.49 ± 0.76	1.53 ± 0.92	0.9977
AUC_(0-t)_	h·μg/mL	14.93 ± 4.82	15.67 ± 12.47	>0.9999	3.01 ± 0.83	4.97 ± 2.39	>0.9999
AUC_(0-∞)_	h·μg/mL	15.19 ± 5.08	13.55 ± 15.16	>0.9999	2.99 ± 0.87	5.55 ± 3.32	>0.9999
AUMC_(0-t)_	h·h·μg/mL	82.28 ± 27.27	42.81 ± 44.28	0.0806	1.56 ± 0.43	2.82 ± 1.39	>0.9999
AUMC_(0-∞)_	h·h·μg/mL	87.96 ± 34.55	44.76 ± 46.34	0.0404	2.35 ± 1.33	8.18 ± 8.21	0.6643
MRT_(0-t)_	h	5.56 ± 0.17	3.82 ± 0.83	>0.9999	0.57 ± 0.04	0.66 ± 0.07	>0.9999
MRT_(0-∞)_	h	5.72 ± 0.38	3.36 ± 0.46	>0.9999	0.75 ± 0.21	1.26 ± 0.73	>0.9999

**Table 8:** Tissue concentrations after oral administration of 28 mg/kg kokusaginine in rats (Data are Mean ± SD, *n* = 4, Equal Sex Ratio).

Tissue	Concentration (μg/mL)				
	0.5 h	2 h	4 h	8 h	10 h
Heart	4.32 ± 3.14	4.89 ± 2.82	5.06 ± 2.99	4.24 ± 4.00	-
Liver	8.32 ± 3.18	7.62 ± 4.24	5.60 ± 4.68	5.08 ± 2.75	2.01 ± 0.00
Spleen	1.70 ± 1.30	1.56 ± 1.23	0.91 ± 0.55	0.63 ± 0.35	0.03 ± 0.00
Lung	2.95 ± 1.08	3.12 ± 1.97	3.35 ± 1.98	1.94 ± 1.11	-
Kidney	3.59 ± 1.53	4.04 ± 2.39	2.80 ± 1.30	2.73 ± 1.72	-
Brain	2.88 ± 1.05	3.28 ± 1.94	2.50 ± 1.41	2.29 ± 1.44	-

**Table 9:** The tissue-plasma partition coefficient (K_p_) calculated by comparing rat AUC_(tissue)_/AUC_(plasma)_ (Data are Mean ± SD, *n* = 4, Equal Sex Ratio).

Tissue	AUC_(0-t)_	K_p_
Plasma	42.87 ± 10.60	-
Heart	37.33 ± 23.45	0.87 ± 0.44
Liver	54.69 ± 11.05	1.29 ± 0.07
Spleen	10.89 ± 4.29	0.25 ± 0.07
Lung	22.14 ± 2.87	0.53 ± 0.08
Kidney	25.60 ± 7.72	0.61 ± 0.15
Brain	23.00 ± 7.59	0.59 ± 0.33

**Table 10:** Comparison of kokusaginine concentration and pharmacokinetic parameters in heart, liver, spleen, lung, kidney and brain of rats at different time points and between different genders (Data are Mean ± SD, *n* = 4, Equal Sex Ratio).

Tissue	Time	Unit	Male Rats	Female Rats	*p* Value	Pharmacokinetic Parameters	Unit	Male Rats	Female Rats	*p* Value
Heart	0.5 h	μg/mL	2.39 ± 0.30	6.25 ± 3.82	>0.9999	T_max_	h	2.00 ± 0.00	5.00 ± 4.24	>0.9999
	2 h		5.71 ± 4.27	4.07 ± 1.73	>0.9999	C_max_	μg/mL	5.71 ± 4.27	7.43 ± 3.02	0.9999
	4 h		1.43 ± 1.38	6.40 ± 2.69	>0.9999	AUC_(0-t)_	h·μg/mL	25.8 ± 20.55	48.86 ± 26.37	0.9989
	8 h		3.05 ± 2.88	5.44 ± 5.84	>0.9999	AUMC_(0-t)_	h·h·μg/mL	87.03 ± 77.73	181.05 ± 121.45	0.0984
	10 h		-	-	-	MRT_(0-t)_	h	3.58 ± 0.64	3.95 ± 0.93	>0.9999
Liver	0.5 h	μg/mL	6.24 ± 0.78	10.42 ± 3.49	>0.9999	T_max_	h	3.00 ± 1.41	1.25 ± 1.06	>0.9999
	2 h		7.91 ± 3.57	7.35 ± 6.40	>0.9999	C_max_	μg/mL	11.03 ± 0.85	12.38 ± 0.71	>0.9999
	4 h		7.52 ± 5.82	3.68 ± 4.14	>0.9999	AUC_(0-t)_	h·μg/mL	56.01 ± 3.73	53.38 ± 18.59	>0.9999
	8 h		4.47 ± 2.75	5.70 ± 3.68	>0.9999	AUMC_(0-t)_	h·h·μg/mL	192.44 ± 13.67	209.31 ± 149.04	>0.9999
	10 h		-	2.01 ± 0.00	>0.9999	MRT_(0-t)_	h	3.75 ± 0.21	3.84 ± 1.47	>0.9999
Spleen	0.5 h	μg/mL	0.79 ± 0.04	2.62 ± 1.31	>0.9999	T_max_	h	3.00 ± 1.41	1.25 ± 1.06	>0.9999
	2 h		1.51 ± 1.22	1.62 ± 1.74	>0.9999	C_max_	μg/mL	1.92 ± 0.64	3.20 ± 0.49	>0.9999
	4 h		1.04 ± 0.60	0.79 ± 0.70	>0.9999	AUC_(0-t)_	h·μg/mL	8.77 ± 0.88	13.01 ± 6.03	>0.9999
	8 h		0.75 ± 0.40	1.73 ± 1.87	>0.9999	AUMC_(0-t)_	h·h·μg/mL	30.03 ± 9.28	56.31 ± 50.37	0.2954
	10 h		-	0.03 ± 0.00	>0.9999	MRT_(0-t)_	h	3.72 ± 0.93	3.91 ± 2.02	>0.9999
Lung	0.5 h	μg/mL	3.12 ± 1.79	2.80 ± 0.43	>0.9999	T_max_	h	2.25 ± 2.47	5.00 ± 1.24	>0.9999
	2 h		2.85 ± 1.66	3.39 ± 2.91	>0.9999	C_max_	μg/mL	4.91 ± 0.75	4.49 ± 1.36	>0.9999
	4 h		3.47 ± 2.79	1.80 ± 1.87	>0.9999	AUC_(0-t)_	h·μg/mL	22.67 ± 1.93	21.61 ± 4.46	>0.9999
	8 h		1.39 ± 0.58	2.49 ± 1.47	>0.9999	AUMC_(0-t)_	h·h·μg/mL	75.37 ± 17.47	74.73 ± 35.46	>0.9999
	10 h		-	-	-	MRT_(0-t)_	h	3.52 ± 0.41	3.81 ± 1.26	>0.9999
Kidney	0.5 h	μg/mL	2.38 ± 0.26	4.81 ± 1.03	>0.9999	T_max_	h	2.00 ± 0.00	1.25 ± 1.06	>0.9999
	2 h		4.38 ± 2.24	3.70 ± 3.42	>0.9999	C_max_	μg/mL	4.38 ± 2.24	5.68 ± 0.63	>0.9999
	4 h		2.06 ± 0.27	2.45 ± 2.60	>0.9999	AUC_(0-t)_	h·μg/mL	22.66 ± 5.85	28.54 ± 10.49	>0.9999
	8 h		2.15 ± 1.00	3.31 ± 2.55	>0.9999	AUMC_(0-t)_	h·h·μg/mL	75.61 ± 20.51	97.58 ± 60.54	0.7992
	10 h		-	-	-	MRT_(0-t)_	h	3.67 ± 0.13	3.67 ± 1.24	>0.9999
Brain	0.5 h	μg/mL	2.01 ± 0.09	3.77 ± 0.46	>0.9999	T_max_	h	2.00 ± 0.00	5.00 ± 4.24	0.9999
	2 h		3.26 ± 1.51	3.31 ± 3.01	>0.9999	C_max_	μg/mL	3.26 ± 1.51	4.84 ± 0.84	>0.9999
	4 h		1.39 ± 0.07	3.61 ± 1.03	>0.9999	AUC_(0-t)_	h·μg/mL	17.10 ± 5.45	28.90 ± 1.93	0.3618
	8 h		1.74 ± 1.05	2.84 ± 1.99	>0.9999	AUMC_(0-t)_	h·h·μg/mL	56.93 ± 21.09	102.18 ± 9.17	< 0.0001
	10 h		-	-	-	MRT_(0-t)_	h	3.66 ± 0.32	3.99 ± 0.95	>0.9999

## Data Availability

Data are contained within the article.

## Supplementary Materials

The following supporting information can be downloaded at: https://www.mdpi.com/article/10.3390/ph17081053/s1, Table S1. Main pharmacokinetic parameters and definitions.
