# Supplementary material for: An Evaluation of Sex-Specific Pharmacokinetics and Bioavailability of Kokusaginine: An In Vitro and In Vivo Investigation
Source: Pharmaceuticals (Basel). 2024 Aug 9;17(8):1053. doi: 10.3390/ph17081053 (PMC11357621; doi:10.3390/ph17081053)
Supplement: Supplementary file 1 [file pharmaceuticals-17-01053-s001.zip › pharmaceuticals-3122303-supplementary.pdf]

Supporting information

An Evaluation of Sex-Specific Pharmacokinetics and Bioavailability of Kokusaginine: An In Vitro and In Vivo Investigation

Kaiqi Shang [1,2,3], Chengyu Ge [1,2,3], Yindi Zhang [1,2,3], Jing Xiao [4], Shao Liu [1,2,3,*] and Yueping Jiang [1,2,3,5,*]

[1] Department of Pharmacy, Xiangya Hospital, Central South University, Changsha 410008, China; 15186814762@163.com (K.S.); gechengyu0408@163.com (C.G.); 228112404@csu.edu.cn (Y.Z.)
[2] National Clinical Research Center for Geriatric Disorders, Xiangya Hospital, Central South University, Changsha 410008, China
[3] The Hunan Institute of Pharmacy Practice and Clinical Research, Changsha 410008, China
[4] Hunan Institute for Drug Control, Changsha 410001, China; 15367917800@189.cn
[5] College of Pharmacy, Changsha Medical University, Changsha 410219, China
* Correspondence: liushao999@csu.edu.cn (S.L.); jiangyueping@csu.edu.cn (Y.J.)

Table S1. Main pharmacokinetic parameters and definitions.

Pharmacokinetic parameters	Definition
K	Estimation of the elimination rate constant (k) from the log-linear final slope of the plasma concentration-time curve (−k/2.303)；
$t_{1/2}$	Plasma terminal elimination half-life，elimination half-life ($t_{1/2}$) was calculated as following Equation (1): $t_{1/2} = -\frac{\text{In}(2)}{k}$
T_{max}	The time to reach peak or maximum plasma concentration, measured value；
C_{max}	The peak or maximum plasma concentrations, measured value；
Vz	Observed Free Volume of Distribution；
CL	Total body clearance of drug；
$AUC_{(0\text{-}t)}$	The theoretical concentration at time zero is deduced from the initial measured concentration, and the area under the concentration-time curve to the last measured concentration is calculated by trapezoidal method.
$AUC_{(0\text{-}\infty)}$	The area under the blood concentration-time curve is obtained from dosing to infinity, calculated according to the following Equation (2)： $AUC_{(0-\infty)} = AUC_{(0-t)} + \frac{C_{last}}{k}$
$AUMC_{(0\text{-}t)}$	From administration to the last measurable product of concentration and time - area under the time curve；
$AUMC_{(0\text{-}\infty)}$	Product of dose concentration and time from dose to infinity - area under time curve；
$MRT_{(0\text{-}t)}$	Refers to the average dwell time from the beginning of drug administration to the last measurable concentration time point,calculated according to the following Equation (8)： $MRT_{(0-t)} = \frac{AUCM_{(0-t)}}{AUC_{(0-t)}}$
$MRT_{(0\text{-}\infty)}$	Refers to the average residence time from the time the drug is administered until it is completely cleared from the body (theoretically infinite moment),calculated according to the following Equation (3)： $MRT_{(0-\infty)} = \frac{AUCM_{(0-\infty)}}{AUC_{(0-\infty)}}$
